# Clinical characteristics and the influence of rs1800470 in patients with Camurati-Engelmann disease

**DOI:** 10.3389/fendo.2022.1041061

**Published:** 2022-10-20

**Authors:** Hanting Liang, Ruizhi Jiajue, Wenting Qi, Wei Liu, Yue Chi, Yan Jiang, Ou Wang, Mei Li, Xiaoping Xing, Weibo Xia

**Affiliations:** Department of Endocrinology, Key Laboratory of Endocrinology, National Commission of Health, State Key Laboratory of Complex Severe and Rare Diseases, Peking Union Medical College Hospital, Chinese Academy of Medical Sciences and Peking Union Medical College, Beijing, China

**Keywords:** Camurati-Engelmann disease, TGFB1, snp, rs1800470, glucocorticoids

## Abstract

**Background:**

Camurati-Engelmann disease (CED) is a sclerosing bone dysplasia caused by transforming growth factor β1 (*TGFB1*) gene variants.

**Objective:**

We aim to summarize the clinical characteristics and the efficacy of glucocorticoids in 14 individuals with CED, and explore the correlation between the phenotype and the SNP of rs1800470 (c.29C>T).

**Methods:**

Clinical, biochemical, radiological, and therapeutic data were collected from 14 patients. DNA was extracted for *TGFB1* variants detection by Sanger sequencing.

**Results:**

The median onset and record age were 3.0 and 16.1 years, respectively. All patients manifested bone pain and decreased subcutaneous fat tissue. Inflammatory markers increased in over 60% of patients, and the median erythrocyte sedimentation rate (ESR) was 1.40 (0.50~3.67) of the upper limit of normal (ULN), and the median high sensitivity C reactive protein (hsCRP) was 1.71 (0.48~12.56) of ULN. There was a positive correlation between ESR and hsCRP (rs=0.806, p=0.003). Both ESR and hsCRP were negatively correlated with the levels of hemoglobin (HGB), calcium, and creatinine, but positively correlated with the level of alkaline phosphatase. Four known variants of *TGFB1* were identified, including p.Tyr171Cys, p.Arg218Cys, p.Arg218His, and p.Cys225Arg. Moreover, 35.7% and 28.6% of them carried the heterozygous and homozygous SNP of c.29C>T, called C/T and T/T groups, respectively, but 35.7% of them were without c.29C>T (C/C group). The onset age, anthropometric data, percentages of different clinical manifestations, and biochemical parameters were comparable among the three groups. But there were increasing trends in levels of HGB and calcium and decreasing trends in ESR and hsCRP among C/C, C/T, and T/T groups in turn. Glucocorticoid improves the two inflammatory markers among CED patients.

**Conclusion:**

The phenotype of CED is highly heterogeneous. There is no clear genotype-phenotype correlation, but it seems to have better trends of biochemical parameters in patients with CED carrying the T allele of rs1800470.

## Introduction

Camurati-Engelmann disease (CED, MIM #131300), also called progressive diaphyseal dysplasia (PDD), is a rare autosomal dominant skeletal disorder that belongs to the group of sclerosing bone dysplasia, the pathogenic gene of which is the transforming growth factor β1 (*TGFB1*) (1, 2). The prevalence of CED is unclear, but more than 300 affected individuals have been described to date (3, 4). The hallmark of the disorder is uneven hyperostosis of the diaphyses of the long bones and skull, extending to both the periosteal surface and the endosteal side of long tubular bones (4). The common clinical characteristics of CED include bone pain mainly involving lower extremities, waddling gaits, proximal muscle weakness, easy fatigability, and thin limbs (4, 5). Other clinical features include nerve compression manifestations, such as hearing loss, visual impairment, facial paralysis, anorexia, joint contractures, hepatosplenomegaly, decreased subcutaneous fat tissue, delayed puberty, and hypogonadism (5, 6).

*TGFB1* gene is located on chromosome 19q13.2 (2, 7), containing seven exons (8), and encoding the precursor protein of TGFB1 with 390 amino acids (4). The precursor consists of a signal peptide, the regulator subunit of latency-associated peptide (LAP), and the active subunit of mature TGFβ1 at the C-terminal (4, 9), and is highly expressed in bones and bone matrix (10). The mature TGFβ1 is a multifunctional protein, which plays a vital role in regulating cell proliferation, differentiation, migration, and apoptosis (4). Most importantly, it mainly regulates the bone-remodeling process and maintains bone homeostasis by promoting bone formation and reducing bone resorption, but the actual condition is rather complicated (11, 12). CED is caused by activation of TGFβ1 (4). The penetrance of CED is unknown, and the phenotype of patients with CED is highly heterogeneous in aspects of clinical manifestations, biochemical abnormalities, and radiological features (13, 14). There is no correlation between the pathogenic variants and the phenotype of CED (14).

Several single nucleoid polymorphisms (SNPs) in the *TGFB1* gene affect the expression of TGFβ1, including rs1800470 of c.29C>T (p.Pro10Leu) (15). The SNP of rs1800470 is located in the signal peptide region, which influences the secretion of TGFβ1 (15). According to data from the 1000 Genomes Project, the allele frequency of c.+29C and c.+29T are 0.4547 and 0.5453 in the *TGFB1* gene. Since the high clinical variability of patients with CED and the moderate frequency of rs1800470 among the general population, we are curious about whether the SNP of rs1800470 is associated with the severity of CED. Therefore, this study aims to analyze the clinical, biochemical, radiological, genetic characteristics, as well as the efficacy of glucocorticoid treatment in a group of individuals with CED. We also explore whether the SNP of rs1800470 affects the phenotype heterogeneity.

## Subjects and methods

### Subjects

A total of 14 CED patients from 12 unrelated families were enrolled. The diagnosis was based on the clinical and radiographic features, and the confirmation of *TGFB1* gene mutation. All subjects or their guardians signed the informed consent. The study conformed to the Declaration of Helsinki and was performed with the approval of the Ethics Committee of Peking Union Medical College Hospital (JS-1689).

### Clinical information collection

We retrospectively reviewed the medical records, including the age of the disorder onset, clinical manifestations, family history, physical examination, treatment, etc. Z scores of height, weight, and body mass index (BMI) in all patients were calculated according to the standardized growth curves for Chinese children (16, 17). Short stature was defined as a Z score of height below 2 standard deviations compared to the mean height of children of the same age and sex. Hepatosplenomegaly was evaluated by physical examination and further certified by abdominal ultrasound. Delayed puberty was defined as the start age of secondary sex characteristics later than 14 years in males and 13 years in females (18), and sex hormone tests were further performed to verify the condition.

### Biochemical measurements

Fasting blood samples of patients were collected. Serum was separated by centrifugation for 10 minutes at 1000×*g*. The routine blood test was measured by using EDTA-anticoagulation peripheral blood. Erythrocyte sedimentation rate (ESR) was detected by an automatic ESR analyzer (VACUETTE). The fresh serum was used to measure the levels of total calcium, phosphate, alkaline phosphatase (ALP), and high sensitivity C reactive protein (hsCRP) by an auto-analyzer (Beckman Coulter AU5800, USA). Total 25-hydroxyvitamin D (T25OHD) and C-terminal cross-linking telopeptide of type I collagen (β-CTX) were measured by the electro-chemiluminescence immunoassay method (Roche Cobas, E601 analyzer, Roche Diagnostics, Switzerland). Levels of parathyroid hormone (PTH), follicle-stimulating hormone, luteinizing hormone, estradiol, and testosterone, were measured by an autoanalyzer (Beckman Coulter DXI800, USA). Reference ranges of all these parameters were obtained from the Department of Laboratory Medicine, PUMCH.

### Radiological assessment

X-rays radiographs were performed for the following skeletal sites: the frontal and lateral cephalometry, humerus, radius, ulna, femur, tibia, and fibula. Two experienced radiologists assessed all these radiographs. Abdominal ultrasound was used to evaluate whether patients have hepatosplenomegaly and the degree of hepatosplenomegaly.

### Genetic tests of *TGFB1* gene

Genomic DNA of all patients was extracted from 200 μl peripheral blood by using a QIAamp DNA Mini Kit (51304, QIAGEN, Germany). The DNA of patients was amplified through polymerase chain reaction (PCR) to detect *TGFB1* gene variants (NM_000660.7). Primers were designed by the online Primer designing tool (https://www.ncbi.nlm.nih.gov/tools/primer-blast/), and the primer information was listed in **Supplementary Materials Table 1**. The 20 µl PCR system consisted of 10 µl of 2×Planta^®^ Flash Master Mix (Dye Plus, P520, Vazyme Biotech Co., Ltd., Nanjing, China), 6 µl of double-distilled water, 2 µl of genomic DNA, and 1 µl of forward and reverse primers (10 µmol/L), respectively. The PCR amplification procedure was as follows: one cycle at 98°C for 30 seconds; 35 cycles at 98°C for 10 seconds, appropriate annealing temperatures for 5 seconds and 72°C for 5 seconds; and one cycle at 72°C for 1 minute followed by storage at 4°C. Then, the product was collected and detected for *TG*FB1 variants by automated DNA sequencing in an ABI DNA sequencer (Model 377, Applied Biosystems, Foster City, CA).

### Statistical analysis

All data were analyzed by SPSS version 24.0. Shapiro-Wilk test was used to determine the normality of continuous variables. The normally distributed data and the abnormally distributed data were described as the mean ± standard deviation (SD) and the median (interquartile range, IQR), respectively. Bivariate analysis was performed to determine the correlation between biochemical parameters, including ESR, hsCRP, hemoglobin (HGB), ALP, calcium, creatinine, T25OHD, PTH, and β-CTX. According to whether patients carried the SNP of c.29C>T in the *TGFB1* gene, they were separated into three groups, which were wildtype (C/C at the 29^th^ allele), heterozygote (C/T at the 29^th^ allele), and homozygote (T/T at the 29^th^ allele) groups. As for continuous variables, the normally distributed data and the abnormally distributed data of the three groups were compared by one-way ANOVA test and Kruskal-Wallis H test, respectively. Classified data were compared by the Fisher’s exact test among the three groups. Biochemical parameters pre and post-glucocorticoid treatment were compared by paired t test and Wilcoxon signed-rank test for the normally distributed data and the abnormally distributed data, respectively. The p value<0.05 was considered a significant difference.

## Results

### General characteristics and clinical manifestations of patients with CED

A total of 14 patients from 12 unrelated families were enrolled, including seven males and seven females, and eleven sporadic cases and three familial cases. The median onset age was 3.0 (1.0~6.0) years old, and the median record age was 16.1 (10.88~22.5) years old. The mean height was 145.1±19.3 cm with a Z score of -1.59±1.47, and the mean weight was 33.9±13.2 kg (Z score: -2.15±1.52). BMI and its Z score were 15.48±3.77 kg/m^2^ and -1.94±1.64, respectively. As **Table 1** showed, all patients manifested bone pain and decreased subcutaneous fat tissue. Genu valgum, waddling gait, enlargement of the mandible, and delayed puberty were common manifestations, accounting for over 75.0%. Muscle weakness, easy fatigability, and hearing loss accounted for more than 50%. Approximately half of these affected individuals had hepatosplenomegaly. Headache, local high skin temperature, impaired vision or visual field defect, and scoliosis were relatively infrequent.

**Table 1:** Clinical manifestations of patients with CED.

Clinical manifestations	Percentage (affected cases/N)
**Bone pain**	100% (14/14)
**Decreased subcutaneous fat tissue**	100% (14/14)
**Genu valgum**	84.6% (11/13)
**Waddling gait**	78.6% (11/14)
**Enlargement of the mandible**	78.6% (11/14)
**Delayed puberty**	75.0% (9/12)
**Muscle weakness**	61.5% (8/13)
**Easy fatigability**	53.8% (7/13)
**Hearing loss**	53.8% (7/13)
**Poor appetite**	46.2% (6/13)
**Hepatosplenomegaly**	46.2% (6/13)
**Exophthalmos**	38.5% (5/13)
**Short stature**	33.3% (4/12)
**Headache**	25.0% (3/12)
**Local high skin temperature**	25.0% (3/12)
**Impaired vision/visual field defect**	21.4% (3/13)
**Scoliosis**	16.7% (2/12)

### Biochemical characteristics and bivariate analysis between biochemical parameters

As shown in **Table 2**, the mean value of hemoglobin (HGB) was 117.7±11.9 g/L, and 50.0% (6/12) of them were anemia. Inflammatory markers increased in more than 60% of patients, including ESR and hsCRP, the median ESR and hsCRP were 1.40 (0.50~3.67) and 1.71 (0.48~12.56) of the upper limit of normal (ULN), respectively. In 63.6% (7/11) of patients, the level of creatinine decreased, with the mean absolute value of 39.0±10.6 μmol/L and 1.05±0.40 of the lower limit of normal (LLN). There were two patients with decreased serum calcium and two patients with elevated serum phosphate. The proportion of vitamin D deficiency (VDD) reached 81.8% (9/11), and PTH slightly increased in two patients due to VDD. As for bone turnover markers, ALP and β-CTX elevated in 58.3% and 90.0% of patients, respectively.

**Table 2:** Key biochemical parameters in patients with CED.

Parameter	Value	Percentage of the parameter increased or decreased
**WBC (×10^9^/L)**	5.73 ± 2.19	–
**HGB (g/L)**	117.7 ± 11.9	↓50.0% (6/12)
**PLT (×10^9^/L)**	288.7 ± 103.1	–
**ESR (mm/h)**	27.0 (10.0~64.0)	↑63.6% (7/11)
**ESR (ULN)**	1.40 (0.50~3.67)	–
**hsCRP (mg/L)**	5.13 (1.43~37.68)	↑69.2% (9/13)
**hsCRP (ULN)**	1.71 (0.48~12.56)	–
**Creatinine (μmol/L)**	39.0 ± 10.6	↓63.6% (7/11)
**Creatinine (LLN)**	1.05 ± 0.40	–
**Calcium (mmol/L)**	2.26 ± 0.13	↓15.4% (2/11)
**Phosphate (mmol/L)**	1.43 ± 0.30	↑15.4% (2/13)
**ALP (U/L)**	264.9 ± 139.0	↑58.3% (7/12)
**T25OHD (ng/ml)**	13.6 ± 7.1	↓81.8% (9/11)
**β-CTX (ng/ml)**	2.13 ± 1.35	↑90.0% (9/10)
**PTH (pg/ml)**	46.4 ± 18.5	↑16.7% (2/12)
**24hUCa (mmol)**	1.23 (0.73~3.02)	–

Reference ranges of all the biochemical parameters were obtained from the central laboratory of PUMCH and were all age/sex/ethnically appropriate. Reference intervals of each parameter: WBC 4.0~10.0 (×10^9^/L); HGB, female 110~150 g/L, male 120~160 g/L; PLT 100~300 (×10^9^/L); ESR 0~20 mm/h; hsCRP 0~3 mg/L; Creatinine 45~84 μmol/L; serum calcium 2.13~2.70 mmol/L; serum phosphate, 4~11 years old 1.19-1.81 mmol/L, 12~15 years old 0.94~1.74 mmol/L, over 15 years old 0.87~1.52 mmol/L; ALP, 0~18 years old 43.0~390.0 U/L, over 18 years old 27.0~107.0 U/L; T25OHD over 20 ng/ml; serum β-CTX 0.21~0.44 ng/ml; PTH 12.0~65.0 pg/ml; 24-hour urinary calcium 1.88~5.63 mmol. WBC, white blood cell; HGB, hemoglobin; PLT, platelets; ESR, erythrocyte sedimentation rate; hsCRP, high sensitivity C reactive protein; ALP, alkaline phosphatase; T25OHD, total 25-hydroxylvitamin D; β-CTX, C-terminal cross-linking telopeptide of type I collagen; PTH, parathyroid hormone; 24hUCa, 24-hour urinary calcium; ULN, upper limit of normal; LLN, lower limit of normal. ↑, above normal upper limit; ↓, below normal lower limit.

**Figure 1:**
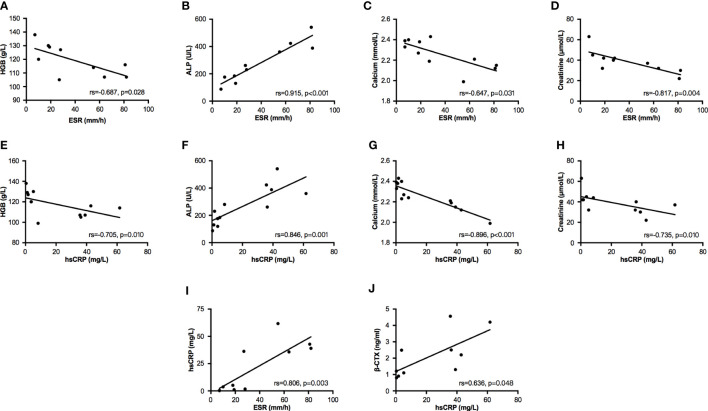
Bivariate correlation analysis between partial biochemical parameters. Bivariate correlations were performed by Spearman analysis. **(A–D)** Relationships between ESR and parameters of HGB, ALP, calcium, and creatinine. **(E–H)** Relationships between hsCRP and parameters of HGB, ALP, calcium, and creatinine. **(I)** The relationship between ESR and hsCRP. **(J)** The relationship between hsCRP and β-CTX. ESR, erythrocyte sedimentation rate; hsCRP, high sensitivity C reactive protein; HGB, hemoglobin; ALP, alkaline phosphatase; β-CTX, C-terminal cross-linking telopeptide of type I collagen.

In bivariate correlation analysis, two inflammatory markers (ESR and hsCRP) had a significantly positive correlation (Spearman coefficient, rs=0.806, p=0.003, **Figure 1I**). Both ESR and hsCRP were negatively correlated with the levels of HGB (ESR: rs=-0.687, p=0.028; hsCRP: rs=-0.705, p=0.010; **Figures 1A, E**), calcium (ESR: rs=-0.647, p=0.031; hsCRP: rs=-0.896, p<0.001; **Figures 1C, G**), and creatinine (ESR: rs=-0.817, p=0.004; hsCRP: rs=-0.735, p=0.010; **Figures 1D, H**), but positively correlated with the level of ALP (ESR: rs=0.915, p<0.001; hsCRP: rs=0.846, p=0.001; **Figures 1B, F**). The level of β-CTX had a positive correlation with hsCRP (rs=0.636, p=0.048, **Figure 1J**), but no correlation with ESR (rs=0.450, p=0.192, **Supplementary Material Table 2**). In addition, T25OHD was negatively associated with levels of ESR (rs=-0.615, p=0.044) and hsCRP (rs=-0.627, p=0.039), and positively correlated with creatinine (rs=0.855, p=0.002, **Supplementary Material Table 2**).

### Radiological characteristics

**Figure 2:**
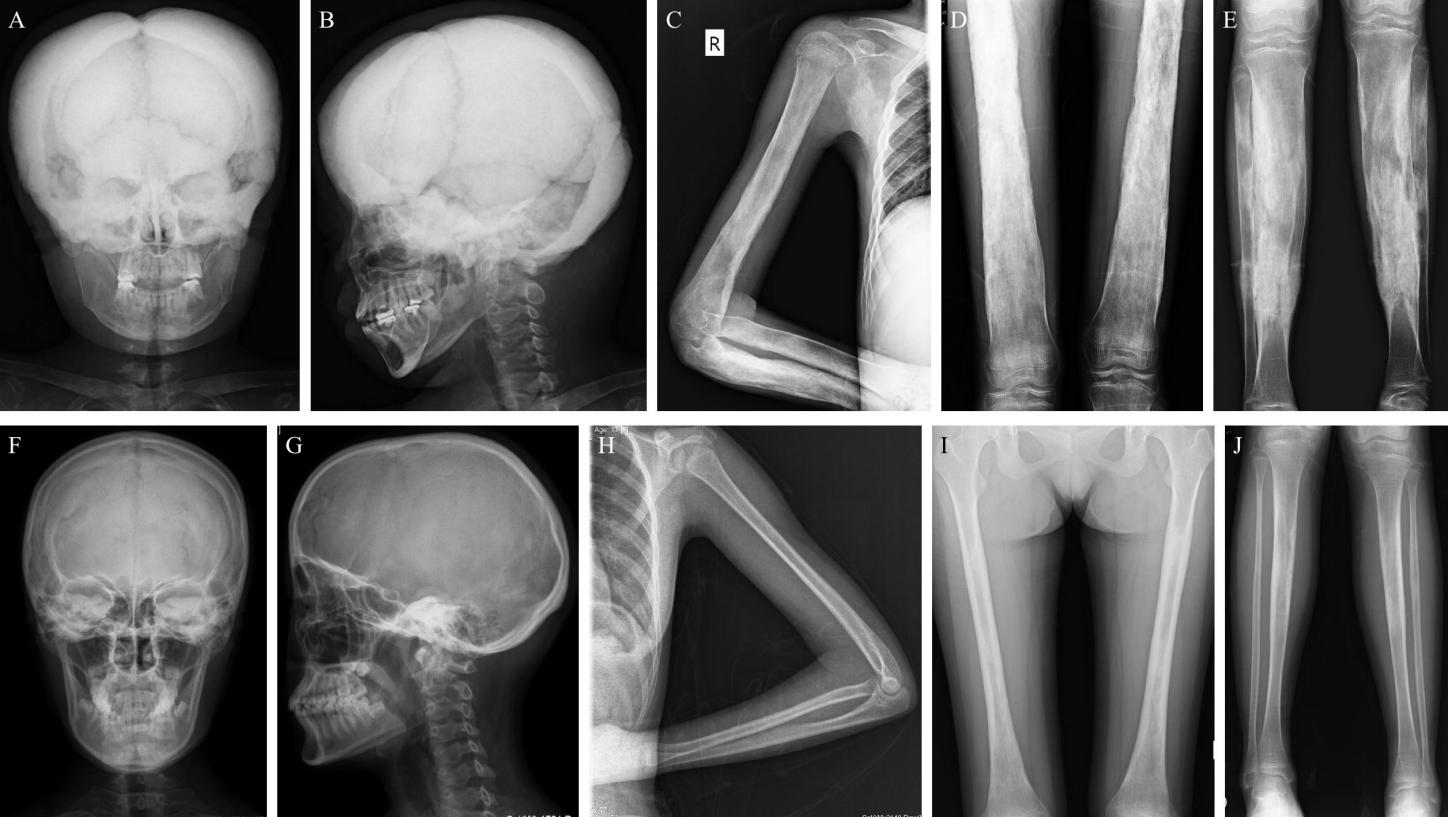
Typical X-rays of patients with CED. **(A–E)** are relatively severe radiographs of an 18 years old female with CED, and **(F–J)** show mild changes in radiographs of patients with CED (**F–I**: female/13 years old; **J**: female/18 years old). **(A, B)** The skull and skull base have severe sclerosis and thickening on the anteroposterior and lateral skull radiographs. **(C–E)** Obvious cortical thickening diaphysis and narrowed medullary canal in the humerus, radius, ulna, femur, tibia, and fibula. **(F, G)** The skull and skull base have no obvious thickening or sclerosis. **(H)** Slight cortical thickening diaphysis and narrowed medullary canal in the ulna but not the humerus or radius. **(I, J)** Mild cortical thickening diaphysis and narrowed medullary canal in the femur and tibia. CED, Camurati-Engelmann disease.

Eleven patients did an X-ray examination. The most common radiological abnormalities were cortical thickening diaphysis and narrowed medullary canal, both of which occurred in all these patients. Another common abnormality was sclerosis of the skull, accounting for 81.8% (9/11), and 63.6% (7/11) of the patients had sclerosis of the skull base. Other less common abnormalities included pelvis deformity, coxa valgus, and osteoporosis in some bones. However, the severity degree of radiological abnormalities varied from patient to patient. As shown in **Figures 2A–E**, the X-ray images of an 18 years old female patient displayed severe sclerosis and thickening on the skull and skull base, predominant cortical thickening diaphysis, and narrowed medullary canal in the humerus, radius, ulna, femur, tibia, and fibula. But some patients had slight radiological changes as **Figures 2F–J** showed, there was no obvious abnormality on the skull and left arm radiographs, and cortical thickening diaphysis could only be found in the focal femur, tibia, and fibula.

### Summary of *TGFB1* gene sequencing

All 14 patients carried with pathogenic variants in the *TGFB1* gene. Four missense mutations were detected, which were c.512A>G (p.Tyr171Cys), c.652C>T (p.Arg218Cys), c.653G>A (p.Arg218His), and c.673T>C (p.Cys225Arg), and the percentage of each mutation was 7.1% (1/14), 71.4% (10/14), 14.3% (2/14), and 7.1% (1/14), respectively. In addition, among all patients, 35.7% (5/14) and 28.6% (4/14) of them carried C/T alleles and T/T alleles of rs1800470, respectively, but 35.7% (5/14) of them carried C/C alleles of rs1800470. The pathogenic variants and SNP information of rs1800470 in the *TGFB1* gene are listed in **Table 3**. Results of Sanger sequencing of all patients are shown in **Supplementary Material Figure 1**.

**Table 3:** Variant information in the *TGFB1* gene of 14 patients with CED.

No.	Affected cases	Pathogenic variant	SNP information of rs1800470
1	1	c.512A>G (p.Tyr171Cys)	c.29C>T (p.Pro10Leu), Het
2	4	c.652C>T (p.Arg218Cys)	C/C, wild type
3	2	c.652C>T (p.Arg218Cys)	c.29C>T (p.Pro10Leu), Het
4	4	c.652C>T (p.Arg218Cys)	c.29C>T (p.Pro10Leu), Homo
5	1	c.653G>A (p.Arg218His)	c.29C>T (p.Pro10Leu), Het
6	1	c.653G>A (p.Arg218His)	c.29C>T (p.Pro10Leu), Homo
7	1	c.673T>C (p.Cys225Arg)	C/C, wild type

SNP of rs1800470: c.29C>T (p.Pro10Leu) in the TGFB1 gene. TGFB1, transforming growth factor β1; CED, Camurati-Engelmann disease; SNP, single nucleotide polymorphism; Het, heterozygote; Homo, homozygote.

### Analysis of relationship between genotype and phenotype

According to whether patients carried the heterozygous or homozygous SNP of c.29C>T in the *TGFB1* gene, they were separated into three groups, which were C/C (C/C at the 29^th^ allele), C/T (C/T at the 29^th^ allele), and T/T (T/T at the 29^th^ allele) groups. The allele frequency of both C and T at this position is 50%. As **Table 4** showed, all parameters had no significant differences among the three groups, including onset age, Z scores of height, weight, and BMI, percentages of different clinical manifestations, biochemical values, etc. Although without significant differences, there were increasing trends in levels of HGB and calcium and decreasing trends in ESR and hsCRP among C/C, C/T, and T/T groups in turn.

**Table 4:** Comparison among CED patients carrying C/C, C/T, and T/T alleles of rs1800470 in the *TGFB1* gene.

	C/C of rs1800470 (n=5)	C/T of rs1800470 (n=4)	T/T of rs1800470 (n=5)	p
**Onset age (years old)**	1.5 (1.0, 4.3)	3.0 (1.0, 15.5)	4.0 (2.5, 8.0)	0.516
**Height Z score**	-1.12 ± 0.84	-1.86 ± 2.04	-1.66 ± 1.49	0.917
**Weight Z score**	-3.08 ± 0.92	-2.01 ± 1.64	-1.71 ± 1.74	0.496
**BMI Z score**	-3.39 ± 1.16	-1.37 ± 1.07	-1.52 ± 1.94	0.219
**Enlargement of the mandible**	100% (5/5)	75.0% (3/4)	60.0% (3/5)	0.451
**Exophthalmos**	50.0% (2/4)	50.0% (2/4)	20.0% (1/5)	0.627
**Hearing loss**	75.0% (3/4)	50.0% (2/4)	40.0% (2/5)	0.790
**Hepatosplenomegaly**	50.0% (2/4)	50.0% (2/4)	40.0% (2/5)	> 0.999
**Delayed puberty**	100% (3/3)	75.0% (3/4)	60.0% (3/5)	0.727
**Headache**	66.7% (2/3)	25.0% (1/4)	0% (0/5)	0.123
**HGB (g/L)**	110.5 (105.5, 118.5)	118.0 (103.3, 113.5)	128.0 (112.0, 129.8)	0.311
**Anemia**	75.0% (3/4)	50.0% (2/4)	25.0% (1/4)	0.766
**ESR (ULN)**	3.64 ± 1.83	2.08 ± 2.87	1.42 ± 1.07	0.315
**Elevated ESR**	100% (3/3)	33.3% (1/3)	60.0% (3/5)	0.455
**hsCRP (ULN)**	12.56 (3.96, 18.68)	2.00 (0.41, 11.39)	0.56 (0.24, 6.81)	0.136
**Elevated hsCRP**	100% (4/4)	75.0% (3/4)	80.0% (4/5)	> 0.999
**Calcium (mmol/L)**	2.14 ± 0.11	2.27 ± 0.12	2.34 ± 0.09	0.055

Height Z score, HGB, and hsCRP were analyzed by Kruskal-Wallis H test, and other parameters were analyzed by one-way ANOVA. Data was depicted as “mean ± standard deviation” or “median (interquartile range)”. CED, Camurati-Engelmann disease; TGFB1, transforming growth factor β1; SNP, single nucleotide polymorphism; BMI, body mass index; HGB, hemoglobin; ESR, erythrocyte sedimentation rate; hsCRP, high sensitivity C reactive protein; ULN, upper limit of normal.

### Treatment monitoring

**Figure 3:**
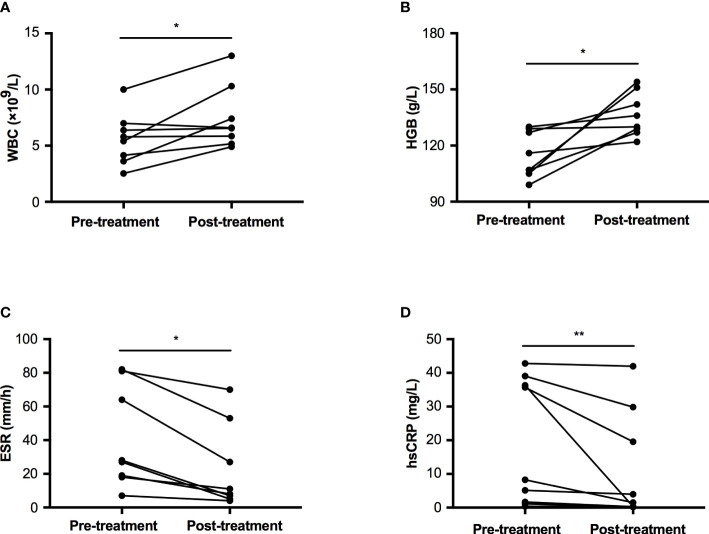
Comparison of WBC, HGB, ESR, and hsCRP pre and post-glucocorticoid treatment in patients with CED. **(A)** The change of WBC pre and post treatment. **(B)** The change of HGB pre and post treatment. **(C)** The change of ESR pre and post treatment. **(D)** The change of hsCRP pre and post treatment. WBC, white blood cell; HGB, hemoglobin; ESR, erythrocyte sedimentation rate; hsCRP, high sensitivity C reactive protein. *p<0.05; **p<0.01.

Nine patients accepted prednisone therapy with a dosage between 10~30 mg per day, the median therapeutic duration was 33.0 months (IQR: 3.5~38.5 months, range: 1~44 months). All of them felt bone pain ameliorated after treatment. The count of white blood cell increased from 5.61±2.32 ×10^9^/L to 7.48±2.80 ×10^9^/L, p=0.029 (**Figure 3A**). The level of HGB was significantly improved from 115.0±12.2 to 136.4±11.6 g/L, p=0.012 (**Figure 3B**). Two inflammatory markers declined significantly (ESR: 27.50 (18.25~76.75) mm/h vs. 9.50 (5.50~46.50) mm/h, p=0.012; hsCRP: 8.25 (1.43~37.68) mg/L vs. 1.52 (0.24~24.71) mg/L, p=0.008, **Figures 3C, D**). However, levels of ALP, calcium, and creatinine had no significant changes pre and post-treatment, which were 247.9±116.9 U/L vs. 234.0±115.5 U/L in ALP, 2.26±0.10 mmol/L vs. 2.32±0.12 mmol/L in calcium, and 35.5±7.7 μmol/L vs. 37.3±7.5 μmol/L in creatinine, respectively.

## Discussion

CED is a quite rare sclerosing bone disorder without a definite prevalence and incidence. The proportion of sporadic cases caused by *de novo* pathogenic variants is also unclear. In this case series, the sporadic cases of CED reached almost 80%. Through typical clinical, biochemical, and radiological characteristics, clinicians can easily draw the clinical diagnosis of CED. Whereas 74% patients have these distinctive features (5), the severity of CED varies from mild to severe, so a definite diagnosis relies on detecting the pathogenic variant in the *TGFB1* gene by the genetic test. Moreover, whether the SNP of rs1800470 is a vital determinant for the severity of CED remains unknown, so we attempt to investigate this issue.

The onset age of most affected individuals is in the second decade of life (14, 19), and the average age of the first symptom onset is 8.7 years old in a large CED cohort (20). In our case series, the median onset age was three years old, which was earlier than other studies described, and it might be due to the high frequency of waddling gait in our cohort, which could be early observed by guardians. The median diagnosis age was 13 years later than the first symptom onset when they came to clinics due to intolerable bone pain or delayed puberty. Similar to other studies, bone pain in the lower extremities, waddling gaits, fatigue, weakness, and decreased muscle bulk and fat tissue are the most common clinical manifestations (5, 13). The skull base manifestations in our study, including hearing loss, exophthalmos, headache, impaired vision, and visual field defect, were more frequent than in the review by Carlson et al. (20), which was possibly due to the smaller sample size of this study. Genu valgum is also a common sign among these patients. The prevalence of hepatosplenomegaly in this study was slightly higher than that in the review by Wallace et al. (37%) (13). There were 75.0% of patients with delayed puberty, it might be attributed to fat hypotrophy (21). Moreover, 1/3 of patients had short stature, we inferred that it might be because their puberty fell far behind their peers.

Markedly increased levels of inflammatory markers in our study revealed the pro-inflammatory effects of TGFβ1 in CED, and the elevated levels of ESR and hsCRP were related to the activity of CED (22, 23). The bone formation marker (ALP) and bone resorption marker (β-CTX) also increased in most patients, and they were positively associated with inflammatory markers, indicating that the bone turnover accelerates with the increased disease activity (23, 24). Moreover, two inflammatory markers were negatively associated with HGB and serum calcium level, which might reflect the disease activity from another aspect. We cautiously inferred that the cortical diaphysis of long bones thickened and the medullary cavity narrowed could aggravate anemia and transfer calcium from circulation to bone under the circumstance of the active disease. Half of the patients in this study were anemia, which was mainly caused by a narrowed medullary cavity in the long bones (4). Over 80% of patients had VDD, and two patients had secondary hyperparathyroidism due to VDD. VDD is a common phenomenon in patients with CED, chronic pain and muscle weakness make patients less willing to engage in outdoor activities so that they are unable to have full sunlight exposure (21).

Radiological changes are also highly variable, that is, some patients have severe sclerosis of whole body bones, while the radiographs are almost normal in other patients. Consistent with the largest review by Janssens et al. (5), in this study, cortical thickening of the diaphysis of the long bones as well as narrowed medullary canal were also the most common radiological abnormalities. The percentage of skull involvement was higher in our study compared to the former two reviews, which were 54% (5) and 56.5% (20), respectively. Symptoms of hearing loss, exophthalmos, headache, impaired vision, and visual field defect have been reported to be associated with the sclerosis of the skull base (20). In addition, local cortical bones are thickening in the diaphysis of long bones, whereas our former study found that the trabecular bone microarchitecture was worse and more inhomogeneous and the cortical bone thickness was thinner than the controls both at the distal radius and the distal tibia (22).

So far, 12 variants of the *TGFB1* gene have been reported, and the corresponding amino acid changes between position 218 to position 225 are identified in over 80% of patients (4). The arginine residue at position 218 is the hotspot mutation, accounting for 60% (5), and the proportion even reached 85.7% in our case series. The pathogenic mechanism caused by variants at different positions is different, but the final effect is the same. Variants of p.L10_12dup and p.L10_13dup at the signal peptide region and p.Y81H at the N-terminal of LAP decrease TGFβ1 secretion, but the accumulation of aberrant TGFβ1 can enhance the transcription of *TGFB1* gene (25). Variants encoded by the second exon at the LAP region may change the electrostatic interactions within the molecule, influencing the conformation of LAP, thus the inhibition of LAP on TGFβ1 bioactivity is impaired (26). Amino acid changes between position 218 to position 225 damage the disulfide bonds, resulting in instability of the dimerization process of LAP, and the TGFβ1 complex is more likely to disassociate and release active TGFβ1 (27, 28). All these different mechanisms can elevate the circulating level of active TGFβ1.

Most studies show no correlation between the genotype and phenotype of CED (5, 14, 26). A previous study investigated the relationship between the SNP of rs1800470 and the phenotype of CED among 24 affected individuals, but the onset age and percentages of pain in limbs, waddling gait, and increased bone density were not different between patients with and without the polymorphism (14). However, in different study populations, the serum TGFβ1 level in subjects with the C/C genotype was significantly or slightly higher than in individuals with the T/T or C/T genotype (29, 30). An *in vitro* study showed that the allele of c.+29C caused an increase in TGFβ1 secretion compared to the allele of c.+29T (31). These results provided the theoretical basis for our initial hypothesis, that was, CED patients with the allele of c.+29T might have a milder phenotype than those with the allele of c.+29C. It is a pity we did not find significant statistical differences in the onset age, percentages of clinical manifestations, and biochemical parameters among patients with C/C, C/T, and T/T alleles due to the limited sample size. Inspiringly, there was a better trend in parameters of HGB, two inflammatory markers, and calcium among patients with T/T alleles.

Consistent with previous studies (5, 6, 21, 23, 32, 33), our study proved that glucocorticoids successfully relieved symptoms and biochemical abnormalities among patients with CED. Loss of follow-up is a problem in this study, which may be because the symptoms such as bone pain relieve automatically with aging in CED patients (19). The value of bisphosphates in treating CED is limited and even associated with severe adverse effects (23). As a novel therapy, the TGF-β type 1 receptor inhibitor (TβR1I) deserves expectation (34). Injecting TβR1I alone ameliorates the symptoms of CED, but the systemic administration causes severe side effects. The conjugate of TβR1I and alendronate can deliver TβR1I to the bone, thus restoring bone turnover, and maintaining the normal bone morphology and remodeling in CED mice (34).

In this study, there are also some limitations. First, clinical manifestations and radiological characteristics were not able to be quantified, which was disadvantageous for analyzing the genotype-phenotype relationship and evaluating the efficacy of glucocorticoids. For example, we did not use the visual analog scale to estimate the degree of bone pain among all patients. Second, the sample size was limited, which was not beneficial for genotype and phenotype analysis, and the data on therapeutic efficacy is so limited that it cannot be compared among the three groups. Third, efficacy evaluation was not precise enough due to different compliance with medication and follow-up among patients. Fourth, the level of serum TGFβ1 was not measured to gain direct evidence of the correlation between the level of circulating TGFβ1 and the SNP of rs1800470. Further mechanism study is needed to confirm the influence of rs1800470 on the disorder.

In conclusion, clinical, biological, and radiological characteristics are highly variable in this cohort of CED. Levels of HGB, ESR, hsCRP, and ALP are vital parameters for evaluating the severity of the disorder. The allele of C or T at rs1800470 has no relationship with the phenotype of CED, but there seems to have better trends of biochemical parameters in patients with CED carrying the T allele of rs1800470. Glucocorticoid treatment is effective for improving bone pain and inflammatory markers in patients with CED.

## Data availability statement

The original contributions presented in the study are included in the article/**Supplementary Materials**. Further inquiries can be directed to the corresponding author.

## Ethics statement

The studies involving human participants were reviewed and approved by the Ethics Committee of Peking Union Medical College Hospital. Written informed consent to participate in this study was provided by the participants’ legal guardian/next of kin.

## Author contributions

WX designed the study and revised the manuscript. HL and RJ collected the blood samples, analyzed the genetic results, and draft the manuscript. HL did the statistical analysis. WQ, WL, YC, YJ, OW, ML, XX, and WX collected clinical information of patients. All authors contributed to the article and approved the submitted version.

## Funding

This study was supported by the National Natural Science Foundation of China (81970757, 81900811), the Chinese National Key Technology R & D Program, Ministry of Science and Technology (2021YFC2501700), and the Chinese Academy of Medical Sciences-CAMS Innovation Fund for Medical Sciences (CIFMS-2021–12M-1–002).

## Acknowledgments

We are so grateful to all of the participants in this study.

## Conflict of interest

The authors declare that the research was conducted in the absence of any commercial or financial relationships that could be construed as a potential conflict of interest.

## Publisher’s note

All claims expressed in this article are solely those of the authors and do not necessarily represent those of their affiliated organizations, or those of the publisher, the editors and the reviewers. Any product that may be evaluated in this article, or claim that may be made by its manufacturer, is not guaranteed or endorsed by the publisher.
