# Supplementary material for: Clinical characteristics and the influence of rs1800470 in patients with Camurati-Engelmann disease
Source: Front Endocrinol (Lausanne). 2022 Oct 20;13:1041061. doi: 10.3389/fendo.2022.1041061 (PMC9631481; doi:10.3389/fendo.2022.1041061)

**Supplementary materials Table 1. Primers for TGFB1 gene sequencing.**

| **Primer** | **Sequence** | **Product length (bp)** | **Annealing temperature (°C)** |
| --- | --- | --- | --- |
| TGFB1-1F | 5’ ATCCCCTATTCAAGACCACCCAC 3’ | 906 | 58 |
| TGFB1-1R | 5’ TCCCCCTATTGCTTGTCTCCCTCT 3’ |  |  |
| TGFB1-2F | 5' CTGTCAGCTCCAAAACTCC 3' | 345 | 56 |
| TGFB1-2R | 5' ACCTTGTAACCAGCCGAC 3' |  |  |
| TGFB1-3F | 5' TGGGTACTGTTGGGGAGGAT 3' | 337 | 62 |
| TGFB1-3R | 5' GGGAGAAACAGGGGTGGG 3' |  |  |
| TGFB1-4F | 5' TGGGGTTTGCTCCTTCCTTC 3’ | 292 | 63 |
| TGFB1-4R | 5' TGTGGGAGTCAGGGGATAGG 3' |  |  |
| TGFB1-5F | 5' CGCCCCACTTATCTATCCCTC 3' | 379 | 62 |
| TGFB1-5R | 5' TCTTACACCCAGACCTCATCCC 3' |  |  |
| TGFB1-6F | 5' GTTATTTTGTATGTTCCAGG 3' | 704 | 55 |
| TGFB1-6R | 5' CTCTGTGGGTCTTCATAGC 3' |  |  |
| TGFB1-7F | 5' TAGAAGATAAGAGAGACCG 3' | 691 | 52 |
| TGFB1-7R | 5' TGCTATGGTGACTGAATG 3' |  |  |

**Supplementary materials Table 2. Bivariate correlation analysis between different biochemical parameters.**

|  |  | HGB (g/L) | ESR (mm/h) | hsCRP (mg/L) | Calcium (mmol/L) | Phosohate (mmol/L) | ALP (U/L) | β-CTX (ng/ml) | T25OHD (ng/ml) | PTH (pg/ml) | Cr (μmol/L) |
| --- | --- | --- | --- | --- | --- | --- | --- | --- | --- | --- | --- |
| HGB (g/L) | rs | 1 | **-0.687** | **-0.705** | 0.538 | -0.109 | **-0.610** | -0.629 | 0.600 | -0.419 | 0.439 |
|  | p |  | **0.028** | **0.010** | 0.071 | 0.736 | **0.035** | 0.070 | 0.067 | 0.199 | 0.177 |
| ESR (mm/h) | rs |  | 1 | **0.806** | **-0.647** | 0.287 | **0.915** | 0.450 | **-0.615** | 0.073 | **-0.817** |
|  | p |  |  | **0.003** | **0.031** | 0.392 | **<0.001** | 0.192 | **0.044** | 0.831 | **0.004** |
| hsCRP (mg/L) | rs |  |  | 1 | **-0.896** | 0.245 | **0.846** | **0.636** | **-0.627** | 0.196 | **-0.735** |
|  | p |  |  |  | **<0.001** | 0.420 | **0.001** | **0.048** | **0.039** | 0.541 | **0.010** |
| Calcium (mmol/L) | rs |  |  |  | 1 | 0.071 | **-0.664** | -0.586 | 0.417 | -0.067 | 0.523 |
|  | p |  |  |  |  | 0.819 | **0.019** | 0.075 | 0.202 | 0.836 | 0.099 |
| Phosohate (mmol/L) | rs |  |  |  |  | 1 | 0.100 | 0.411 | -0.595 | 0.234 | -0.215 |
|  | p |  |  |  |  |  | 0.757 | 0.237 | 0.054 | 0.465 | 0.526 |
| ALP (U/L) | rs |  |  |  |  |  | 1 | 0.503 | -0.584 | 0.304 | **-0.806** |
|  | p |  |  |  |  |  |  | 0.168 | 0.076 | 0.364 | **0.003** |
| β-CTX (ng/ml) | rs |  |  |  |  |  |  | 1 | -0.446 | 0.564 | -0.309 |
|  | p |  |  |  |  |  |  |  | 0.197 | 0.089 | 0.419 |
| T25OHD (ng/ml) | rs |  |  |  |  |  |  |  | 1 | -0.541 | **0.855** |
|  | p |  |  |  |  |  |  |  |  | 0.085 | **0.002** |
| PTH (pg/ml) | rs |  |  |  |  |  |  |  |  | 1 | -0.329 |
|  | p |  |  |  |  |  |  |  |  |  | 0.323 |
| Cr (μmol/L) | rs |  |  |  |  |  |  |  |  |  | 1 |
|  | p |  |  |  |  |  |  |  |  |  |  |

The relationship between ESR or hsCRP and other parameters were analyzed by Spearman analysis, while other parameters were analysed by Pearson analysis. Abbreviations: HGB, hemoglobin; ESR, erythrocyte sedimentation rate; hsCRP, high sensitivity C reactive protein; ALP, alkaline phosphatase; β-CTX, C-terminal cross-linking telopeptide of type I collagen; T25OHD, total 25-hydroxylvitamin D; PTH, parathyroid hormone; Cr, creatinine.

**Figure 1. Sanger sequencing of TGFB1 gene in 14 patients with CED.**


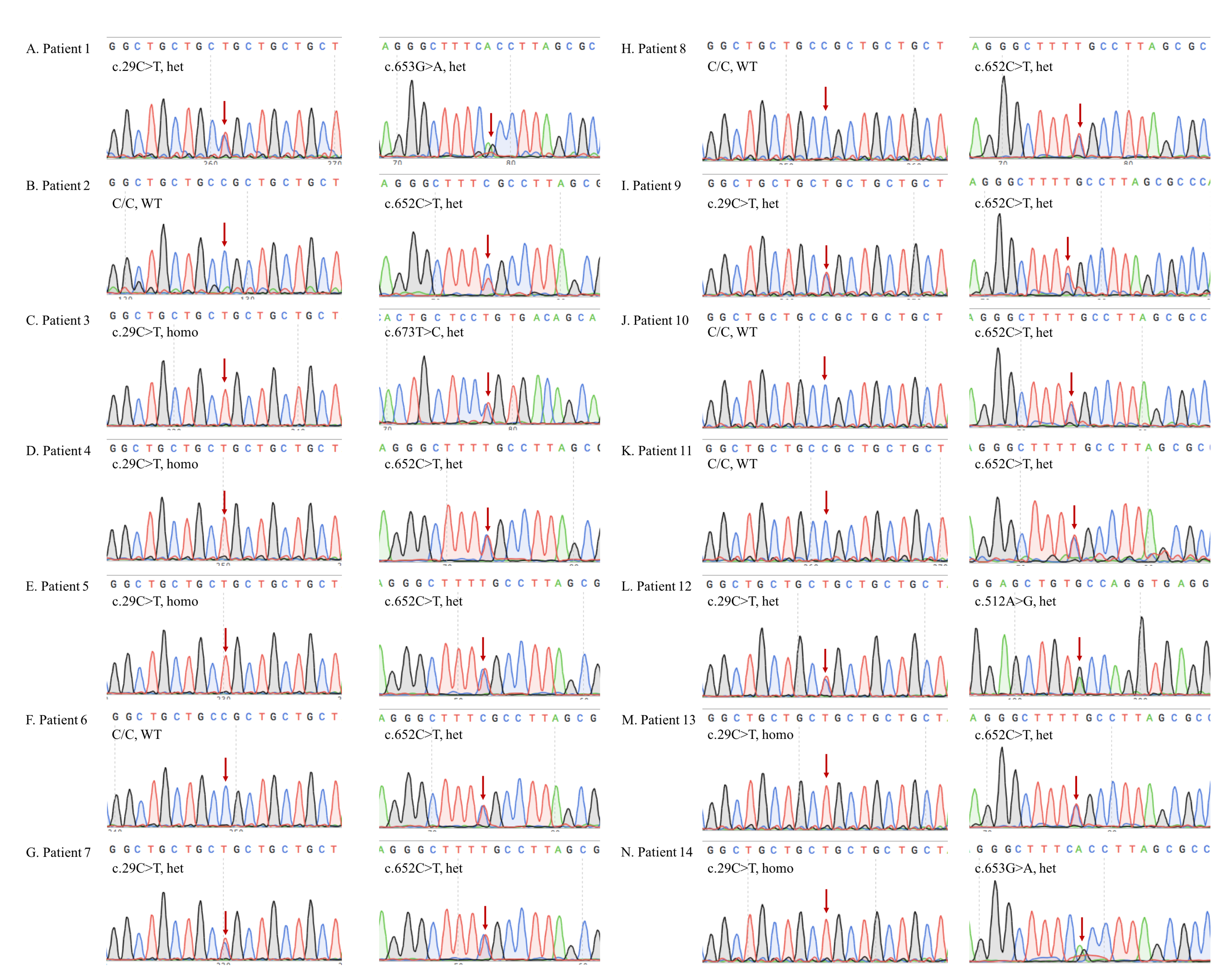

Supplement: Supplementary file 1 [file DataSheet_1.docx]
